# Can high-frequency ultrasound combined computed tomography accurately diagnose thyroid tumor? A protocol of systematic review and meta-analysis

**DOI:** 10.1097/MD.0000000000020180

**Published:** 2020-05-15

**Authors:** Lin-hua Zhang, Gang Chen, Wei Tao

**Affiliations:** a Department of Ultrasound; b Department of Radiology, Ningbo Municipal Hospital of TCM, Ningbo, China.

**Keywords:** computed tomography, high-frequency ultrasound, sensitivity, specificity, thyroid tumor

## Abstract

**Background:**

Previous clinical studies have reported that clinical value of high-frequency ultrasound combined computed tomography (HFUCT) is used for diagnosis of thyroid tumor (TT). However, no study has investigated this topic systematically. Therefore, this study will evaluate the clinical value of HFUCT for the diagnosis of TT.

**Methods:**

We will search the databases of Cochrane Library, EMBASE, PUBMED, SCOPUS, Web of Science, OpenGrey, Cumulative Index to Nursing and Allied Health Literature, Allied and Complementary Medicine Database, and China National Knowledge Infrastructure from any time period published to the present. We will consider all case-controlled studies that assessed the clinical value of HFUCT for diagnosis of TT. Two authors will independently scan titles and abstracts to check eligible studies, followed by full-text read. We will extract data and assess study quality using Quality Assessment of Diagnostic Accuracy Studies tool. RevMan 5.3 software will be utilized for data pooling and statistical analysis.

**Results:**

This study will be performed to assess the clinical value of HFUCT for the diagnosis of TT, and will provide an evidence-based synthesis for clinical application and further study.

**Conclusion:**

Summary of this study will provide the latest evidence to determine whether HFUCT can be used for TT diagnosis accurately.

**Study registration:**

INPLASY202040022.

## 1 Introduction

Thyroid tumor (TT) is a very common disease, which consists of benign tumors and malignant tumors (thyroid cancer).^[1,2,3,4,5]^ It increases with age and is present in almost 10% of adult population,^[5]^ in which, 95% of TT is benign, and 5% of it is malignant.^[5]^ The differential diagnosis of TT is very crucial for the clinical practice.^[6,7,8]^ Thus, it is very important to diagnose TT at early stage.^[9,10,11]^

Previous studies have reported that high-frequency ultrasound combined computed tomography (HFUCT) can help to diagnose TT.^[12,13,14,15,16,17,18]^ However, no study has been undertaken systematically to examine the clinical value of HFUCT for the diagnosis of TT. Therefore, this systematic review will examine the clinical value of HFUCT for TT diagnosis.

## 2 Methods

### 2.1 Objective

This study aims to check the clinical value of HFUCT for the diagnosis of TT.

### 2.2 Study registration

We have registered this study on INPLASY202040022. We will report the results of this study according to the guideline of Preferred Reporting Items for Systematic Reviews and Meta-Analysis Protocol statement.^[19]^

### 2.3 Eligible criteria for including studies

#### 2.3.1 Type of studies

We will only consider case-controlled studies on assessing the clinical value of HFUCT for the diagnosis of TT. However, we will exclude any other studies, such as animal studies, case studies, and nonclinical studies.

#### 2.3.2 Type of participants

We will include studies of reporting patients with histological-proven TT.

#### 2.3.3 Type of index test

Index test: We will include studies using HFUCT for the diagnosis of TT in the experimental group.

Reference test: We will include patients with histological-proven TT in the control group.

#### 2.3.4 Type of outcome measurements

The primary outcomes are sensitivity and specificity. The secondary outcomes are positive likelihood ratio, negative likelihood ratio, and diagnostic odds ratio.

### 2.4 Information sources and search strategy

#### 2.4.1 Electronic searches

We will include any types of studies on assessing the clinical value of HFUCT for diagnosis of TT from any time period published to the present from the following databases: Cochrane Library, EMBASE, PUBMED, SCOPUS, Web of Science, OpenGrey, Cumulative Index to Nursing and Allied Health Literature, Allied and Complementary Medicine Database, and China National Knowledge Infrastructure. No limitations of language and publication status will be imposed. We will present search strategy for Cochrane Library in Table 1. We will also adapt similar search strategies to other electronic databases.

**Table 1:**
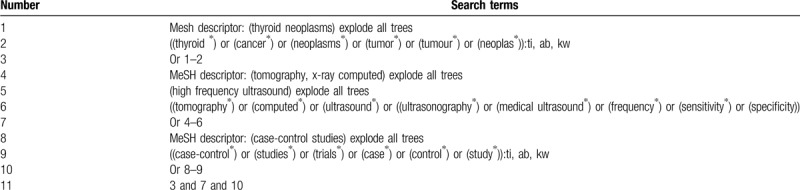
Search strategy of Cochrane Library.

#### 2.4.2 Other resources

We will also check reference lists of other relevant reviews, clinical trial registry, and conference proceedings.

### 2.5 Data records and analysis

#### 2.5.1 Selection process of studies

We will export all citations from the searched resources to the Endnote 7.0, and we will remove any duplicated studies. Two authors will independently review all titles and abstracts to check whether they meet the eligibility criteria for inclusion, and all irrelevant studies will be excluded. Then, full-text articles for studies that fulfill the review criteria will be obtained and judged. The reasons for all excluded studies will be noted. The whole process of study selection will be shown in a flowchart. Any discrepancies at either the initial or full-text screening between 2 authors will be solved or consulted by a third author through discussion when necessary.

#### 2.5.2 Data collection and management

Two authors will independently extract information from each selected study based on the previous designed sheet for data extraction. If there are any discrepancies between 2 authors, a third experienced author will be consulted to solve the different opinions. We will extract the following data: title, first author, publication time, location, patient characteristics, inclusion and exclusion criteria, study method, index test, refer test, outcomes, and funding information. If any insufficient information occurs, we will contact corresponding authors to inquire those data.

### 2.6 Study quality assessment

We will use Quality Assessment of Diagnostic Accuracy Studies tool to assess study quality for each included study.^[20]^ This tool is utilized for evaluating diagnostic accuracy for all included studies. Two authors will independently assess the study quality, and any divergences between 2 authors will be consulted by a third author to make final decision.

### 2.7 Statistical analysis

We will use RevMan V.5.3 software to synthesize the data and to perform data analysis.

We will calculate outcome data using descriptive statistics and 95% confidence intervals.

*I*^*2*^ statistic will be used to check the degree of statistical heterogeneity among included studies with the guide judgments: *I*^*2*^ ≤ 50% indicating low heterogeneity, whereas *I*^*2*^ > 50% exerting obvious heterogeneity. We will pool the data using a fixed-effect model, and we will consider performing a meta-analysis to calculate pooled effect of outcome measurements if those eligible studies are sufficiently homogeneous (*I*^*2*^ ≤ 50%). Otherwise, a random-effect model will be used and a subgroup analysis will be carried out to identify the reasons that may cause obvious heterogeneity.

### 2.8 Additional analysis

#### 2.8.1 Subgroup analysis

We will operate a subgroup analysis based on the characteristics of different study or patient, comparators, and outcomes.

#### 2.8.2 Sensitivity analysis

We will plan to perform a sensitivity analysis by removing low quality studies to check the robustness of outcome results.

#### 2.8.3 Reporting bias

We will check reporting bias using funnel plots and associated regression tests if necessary.^[21]^

### 2.9 Ethics and dissemination

This study does not need ethical approval because it will not analyze individual patient data. The results of this study will be submitted on a peer-reviewed journal.

## 3 Discussion

This study will provide an up-to-date evidence to examine the clinical value of HFUCT for the diagnosis of TT through primary and secondary outcomes assessment. To our best knowledge, this is the first study to focus on the validity of HFUCT diagnoses in TT. This study will summarize the most recent evidence on the clinical value of HFUCT for the diagnosis of TT. Its results may provide useful and up-to-date findings to inform researchers on the validity of using HFUCT in the future research.

## Author contributions

**Conceptualization:** Lin-hua Zhang, Gang Chen, Wei Tao.

**Data curation:** Gang Chen, Wei Tao.

**Formal analysis:** Lin-hua Zhang, Gang Chen, Wei Tao.

**Funding acquisition:** Wei Tao.

**Investigation:** Wei Tao.

**Methodology:** Lin-hua Zhang, Gang Chen.

**Project administration:** Wei Tao.

**Resources:** Lin-hua Zhang, Gang Chen.

**Software:** Lin-hua Zhang, Gang Chen.

**Supervision:** Wei Tao.

**Validation:** Lin-hua Zhang, Gang Chen, Wei Tao.

**Visualization:** Lin-hua Zhang, Gang Chen, Wei Tao.

**Writing – original draft:** Lin-hua Zhang, Gang Chen, Wei Tao.

**Writing – review & editing:** Lin-hua Zhang, Gang Chen, Wei Tao.
